# The prevalence of psychiatric disease in the significant others of patients with known mood and anxious disease

**DOI:** 10.1186/1745-0179-1-12

**Published:** 2005-08-02

**Authors:** Giuseppe Tavormina, Salvatore Corea, Antonio Citron

**Affiliations:** 1 President of "Psychiatric Studies Centre" ("Cen.Stu.Psi."), piazza Portici, 11, 25050 Provaglio di Iseo (BS), Italy; 2 Consultant of Psychiatric Communities "Sol.Co.", Bergamo, Italy; 3 Mental Health Dept., Hospital of Castelfranco Veneto (TV), Italy

**Keywords:** significant others, mood spectrum, anxious spectrum, life events, stress

## Abstract

**Background -:**

Information about the Significant Others (S.O.) of 530 patients with mood and anxious spectrum disorders has been tabulated in this multicentre, retrospective, clinical observational study in order to learn the prevalence of the same mood and/or anxious spectrum diseases in the S.O. of the patients.

**Methods -:**

The 530 outpatients (of age range from 18 to 70 years) with mood and anxious spectrum disorders have been treated by the authors, observed for a seven year period (from January 1995 until May 2003). The patients live in 16 different Italian provinces, but are predominantly from Lombardia and Veneto.

Mood disease (includes substance abuse) was present in 72% of the patients and anxious disease was present in 28% (DSM-IV diagnoses based upon clinical interviews).

The S.O. (various heterosexual long-term relationships) of each patient was interviewed for this study to establish a DSM-IV diagnosis of any psychiatric disease that might be present.

In cases in which the patient had no S.O. or in which information about the S.O. was unavailable, that information was collected. As data was collected, 10 item report was completed for each patient and the respective S.O.

**Results -:**

Patients had an S.O. with a similar mental disease to their own in 41% of cases; only 16% of the patients chose their S.O. with no mental disease; 18% of the patients did not have any S.O. and in 26% of the cases the health of the S.O. was unknown.

**Conclusion -:**

In this multicentre, retrospective, clinical observational study, the corresponding Significant Others of 530 patients with mood and anxious spectrum disorders presented with a high percentage of similar disease to the patients. These findings suggest that it may be appropriate to counsel our patients with these diseases to encourage their respective S.O. to undergo a psychiatric evaluation for possible treatable disease: the first objective of an S.O. is preventive care, secondarily the well-being of the partner may improve the treatment outcomes for the patient.

Furthermore, eventual studies could demonstrate whether the disease of S.O. precedes couple life (therefore a pre-existent cognitive functioning set out for the partner's choice) and whether it might stem from a difficult interpersonal relationship or chronic stress reaction to a life event.

## Background

Information about the Significant Others (S.O.) of 530 patients with mood and anxious spectrum disorders has been tabulated in this multicentre, retrospective, clinical observational study in order to learn the prevalence of the same mood and/or anxious spectrum diseases in the S.O. of the patients.

## Methods

The 530 outpatients (of age range from 18 to 70 years) with mood and anxious spectrum disorders have been treated by the authors, observed for a seven year period (from January 1995 until May 2003). [5]. The patients live in 16 different Italian provinces, but are predominantly from Lombardia and Veneto. [6].

Mood disease (including substance abuse) was present in 72% of the patients and anxious disease was present in 28% (DSM-IV diagnoses based upon clinical interviews). [1].

The S.O. (various heterosexual long-term relationships) of each patient was interviewed for this study to establish a DSM-IV diagnosis of any psychiatric disease that might be present. Psychiatric diagnoses were considered to be either "certain" or "probable" for the S.O. The opinions of "certain diagnosis" of S.O., "probable diagnosis" and "any mental disease" were derived from direct knowledge of the S.O. or from the family and personal anamnestic data taken from the patients. In cases in which the patient had no S.O. or in which information about the S.O. was unavailable, that information was collected as detailed below.

As data was collected the following 10 item report was completed for each patient and the respective S.O.:

1- Patient diagnosis: mood spectrum;

2- Patient diagnosis: anxious spectrum;

3- Certain diagnosis of S.O.: mood spectrum;

4- Certain diagnosis of S.O.: anxious spectrum;

5- Probable diagnosis of S.O.: mood spectrum;

6- Probable diagnosis of S.O.: anxious spectrum;

7- Total diagnoses of S.O. (certain + probable);

8- S.O. without any mental disease;

9- Unknown health of S.O.;

10- No S.O.

## Results

Patients had an S.O. with a similar mental disease to their own in 41% (217/530 pt.) of cases (percentage data obtained from the item n° 7). Only 16% (85/530 pt.) of the patients chose their S.O. with no mental disease (percentage data obtained from the item n° 8).

18% (95/530 pt.) of the patients did not have any S.O. (item 10). In 26% (138/530 pt.) of the cases the health of the S.O. was unknown (item 9).

The sum of patients of items 9 and 10 together (44%) led us to recognize another relationship: if we considered only all the patients with known health of the S.O., we found that 72% of patients had an S.O. with a similar disease to their own.

The Table 1 summarizes all the items and the respective percentages for the total data.

**Table 1:** Summarise of all followed items with obtained percentage

1 – *Patient diagnosis: mood spectrum*	2 – *Patient diagnosis: anxious spectrum*
383 patients (72%)	147 patients (28%)
3 – *Certain diagn. of S.O.: mood spectr.*	4 – *Certain diagn. of S.O.: anxious spectr.*
80 (16%)	35 (6%)
5 – *Probable diagn. of S.O.: mood spect.*	6-*Probable diagn. of S.O.: anxious spect.*
62 (12%)	39 (7%)
7-*Tot. diagn. of S.O. (certain + probable)*	8 – *Any mental disease of S.O.*
216 (40%)	83 (16%)
9 – *Unknown health of S.O.*	10 – *No Significant Others*
138 (26%)	93 patients (18%)

Summarise of all followed items with obtained percentage

## Conclusion

In this multicentre, retrospective, clinical observational study, the corresponding Significant Others of 530 patients with mood and anxious spectrum disorders presented with a high percentage of similar disease to the patients. These findings suggest that it may be appropriate to counsel our patients with these diseases to encourage their respective S.O. to undergo a psychiatric evaluation for possible treatable disease: the first objective of an S.O. is preventive care, secondarily the well-being of the partner may improve the treatment outcomes for the patient. [2,4].

Furthermore, the above mentioned results can also highlight a possible new direction for psychodynamic evaluation of couples. Eventual studies could demonstrate whether the disease of S.O. precedes couple life (therefore a pre-existent cognitive functioning set out for the partner's choice) and whether it might stem from a difficult interpersonal relationship or chronic stress reaction to a life event. [3].
